# Enhancing Wound Healing: A Novel Topical Emulsion Combining CW49 Peptide and Lavender Essential Oil for Accelerated Regeneration and Antibacterial Protection

**DOI:** 10.3390/pharmaceutics15061739

**Published:** 2023-06-15

**Authors:** Valentina Jaramillo, Erika Díaz, Laura N. Muñoz, Andrés Fernando González-Barrios, Jader Rodríguez-Cortina, Juan C. Cruz, Carolina Muñoz-Camargo

**Affiliations:** 1 Department of Biomedical Engineering, Universidad de los Andes, Bogotá 111711, Colombia; v.jaramillom@uniandes.edu.co (V.J.); ea.diazr@uniandes.edu.co (E.D.);; 2 Department of Chemical and Food Engineering, Universidad de los Andes, Bogotá 111711, Colombia; 3 Corporación Colombiana de Investigación Agropecuaria—AGROSAVIA, Centro de Investigación Tibaitatá, Mosquera 250047, Colombia

**Keywords:** wound healing, lavender essential oil, CW49 peptide, antibacterial activity, topical treatment

## Abstract

Wound healing is a complex process involving blood cells, extracellular matrix, and parenchymal cells. Research on biomimetics in amphibian skin has identified the CW49 peptide from *Odorrana grahami*, which has been demonstrated to promote wound regeneration. Additionally, lavender essential oil exhibits anti-inflammatory and antibacterial activities. Given these considerations, we propose an innovative emulsion that combines the CW49 peptide with lavender oil. This novel formulation could serve as a potent topical treatment, potentially fostering the regeneration of damaged tissues and providing robust antibacterial protection for skin wounds. This study investigates the physicochemical properties, biocompatibility, and in vitro regenerative capacity of the active components and the emulsion. The results show that the emulsion possesses appropriate rheological characteristics for topical application. Both the CW49 peptide and lavender oil exhibit high viability in human keratinocytes, indicating their biocompatibility. The emulsion induces hemolysis and platelet aggregation, an expected behavior for such topical treatments. Furthermore, the lavender-oil emulsion demonstrates antibacterial activity against both Gram-positive and Gram-negative bacterial strains. Finally, the regenerative potential of the emulsion and its active components is confirmed in a 2D wound model using human keratinocytes. In conclusion, the formulated emulsion, which combines the CW49 peptide and lavender oil, shows great promise as a topical treatment for wound healing. Further research is needed to validate these findings in more advanced in vitro models and in vivo settings, potentially leading to improved wound-care management and novel therapeutic options for patients with skin injuries.

## 1. Introduction

The skin acts as a barrier to protect the body against microorganisms, damage from the environment, and dehydration. It also regulates temperature, functions as a sensor organ, and synthesizes vitamin D [1,2,3]. The skin is composed of three layers, the hypodermis, the dermis, and the epidermis. The epidermis outer layer is composed of keratinocyte cells. It is divided into five strata where the proliferation process occurs from the basal to the cornified stratum [3,4,5]. Abrasion, laceration, or avulsion can cause wounds that disrupt the skin’s normal structure and functions. They can be classified as either acute or chronic [6,7]. While acute wounds heal normally, chronic wounds can experience tissue damage that lasts longer [2]. Chronic wounds can lead to infection and other diseases such as diabetes or venous ulcers. They also have a longer healing time of up to several weeks [8]. Wound healing involves blood cells, extracellular matrix, and epidermal cells. It is complex and dynamic and includes four stages: hemostasis (hemorrhage), inflammation, proliferation, and remodeling.

In 2014, global health system expenditures on wound care reached USD 2.5 billion, with projections rising to USD 3.5 billion in 2022 [7,9]. In the United States, chronic wounds affect 6 million people, generating an annual cost of USD 25 million [10]. Current treatments include negative pressure therapies, scaffolds, hydrocolloid dressings, foams, and biomembranes [11]. Amongst various treatment modalities, emulsions have gained prominence for wound closure. These versatile formulations can encapsulate both aqueous and oleaginous ingredients, and their rheological properties can be finely tuned. Numerous studies have explored the role of emulsions as topical dermatological vehicles, focusing on their impact on drug release and absorption. A diverse range of compounds has been incorporated into these emulsions, including naturally derived substances such as essential oils extracted from *Allium sativum*, *Zingiber officinale*, *Melaleuca alternifolia*, *Rosmarinus officinalis*, and *Thymus vulgaris*. They also contain antimicrobial peptides for wound infections and other agents commonly used for wound closure, such as silver sulfadiazine and dexpanthenol [12,13,14,15,16].

Around 75% of all pharmacological molecules currently in use are derived from natural sources [17]. This has prompted ongoing research for new drug candidates. Combining different properties or sources, such as animal-derived molecules or plant extracts, is a common strategy to create synergistic results. For example, amphibians are known for their defense mechanisms that are based on antimicrobial or host-defense proteins [17]. These mechanisms have a wide spectrum of activity and exhibit a variety of mechanisms of action, including immunomodulatory, regenerative, and antimicrobial effects [18]. The CW49 peptide was discovered in the skin of *Odorrana grhami*, a frog. It has been reported to be wound-healing in murine models and is a promising candidate to develop regenerative treatments [19,20,21]. The CW49 peptide comprises 11 amino acids (APFRMGICTTN) and exhibits a total net charge of +1, with a Boman index of 1.12 kcal/mol.

Because of their powerful antiseptic and healing properties, plant-based extracts such as Aloe and Cannabis have been gaining popularity in natural therapies [22,23,24]. Essential oils of calendula and lavender have been used to treat skin conditions such as cuts, burns, and wounds. Lavender essential oil is extracted from *Lavandula angustifolia* flowers by steam distillation. It promotes skin healing and has anti-inflammatory, antibacterial, and analgesic properties [25,26,27]. The essential oil is primarily composed of monoterpenoids, such as (-)-linalool, linalyl acetate, 1,8-cineole, and p-cimeno [28]. Both in vitro and in vivo studies have demonstrated the beneficial effects of lavender-oil application. Notably, it has been found to bolster collagen production and foster fibroblast maturation. This coincides with an elevated expression of the transforming growth factor-beta (TGF-β) [26,29,30]. Plant-based extracts have some drawbacks such as volatility, low stability, and low dose compatibility that make them difficult to apply directly to the skin. These extracts are also reported to speed up the formation of granular tissue in the early stages of wound healing [26,29].

The incorporation of lavender essential oil and the CW49 peptide into an emulsion offers a potential alternative to high-cost treatments in wound healing due to their combined properties. Emulsions can address the afford mentioned limitations of lavender essential oil and CW49 peptide by integrating hydrophilic and lipophilic compounds, enhancing the bioactive components’ stability, and promoting their interaction with the skin. This study aims to develop stable emulsions based on lavender oil–CW49 peptide and evaluate their physicochemical and biological properties, as well as their regenerative capacity in 2D monolayers of human keratinocytes. This research is essential for exploring the potential of these emulsions as an in vitro wound-healing treatment in the context of a wound-healing model, leveraging the antibacterial activity of lavender essential oil and the promising properties of the CW49 peptide for topical treatment.

## 2. Materials and Methods

### 2.1. Materials

The lavender essential oil of *Lavandula angustifolia* was purchased from Naturales Casvior (Bogotá, Colombia). Mineral oil (99%), Tween 20^®^ (Polysorbate 20) (GC grade), Span 80^®^ (Sorbitan Monooleate) (GC grade), Thrombin, and Triton X-100 (laboratory grade) were purchased from Sigma-Aldrich (Milwaukee, WI, USA). Carbopol (poly(acrylic-acid)) (99%), 3-(4,5-dimethylthiazol-2-yl)-2,5-diphenyltetrazolium bromide (MTT), and dimethyl sulfoxide (DMSO) were purchased from Sigma-Aldrich (St. Louis, MO, USA). Triethanolamine (99%) was obtained from PanReac AppliChem (Barcelona, Spain). The Mueller Hinton agar for microbiological testing was acquired from Merl-Millipore (Darmstadt, Germany). The bacteria strains were *Staphylococcus aureus* (*S. aureus*) (ATCC 23235) and *Escherichia coli* (*E. coli*) (ATCC 25922). The CW49 peptide was purchased from GL Biochem Ltd. (Shanghai, China). The human keratinocytes were HaCaT (CVCL 0038). Dulbecco’s modified Eagle’s medium (DMEM) and fetal bovine serum (FBS) were purchased from Biowest (Kansas City, MO, USA).

### 2.2. Methods

#### 2.2.1. Chemical Composition of the Lavender Essential Oil with Gas Chromatography—Coupled to Mass Spectrometry (GC-MS)

The total composition of the lavender essential oil was analyzed by GC-MS using capillary columns (Norm ISO 7609-1985(E)) and it was performed by the CROM-MASS Laboratory at Universidad Industrial de Santander (UIS). The analysis was carried out in an Agilent Technologies 6890 gas-chromatography instrument equipped with a DB-5MS column (J & W Scientific, Folsom, CA, USA) with 5%-Ph-PDMS stationary phase (5% phenyl polydimethylsiloxane, 60 m × 0.25 mm, film thickness 0.25 μm) and coupled with an AT 5973N mass-selective detector of the same company. The GC was equipped with a split injector with an injection volume of 2 μL and a split ratio of ca. 30:1. The MS was operated in full-scan mode, and a detector voltage of 70 eV. The sample was diluted with dichloromethane. The reference standard was a certificate mix of hydrocarbons C_6_–C_25_ (AccuStandar, New Haven, CN, USA).

#### 2.2.2. Emulsions Preparation

Three oil-in-water (O/W) emulsions were prepared following the methodology developed by Muñoz et al. [31]: a control emulsion (without lavender oil and CW49 peptide), a lavender emulsion (with 0.25% *w*/*w* of lavender oil), and a lavender with the CW49-peptide emulsion (with 0.25% *w*/*w* of lavender oil and 20 μg/mL). The emulsions were prepared at a ratio of 10:90% *w*/*w*, with a surfactant ratio of 60:40%, which corresponds to the 4% of total weight, 0.3% *w*/*w* of Carbopol was added as a thickener, and triethanolamine at a 0.6% *w*/*w* concentration. The final composition of the emulsions is summarized in Table 1. Briefly, the aqueous phase without Carbopol was homogenized with a Hei-TORQUE Precision 200 stirrer (Heidolph Instruments, Schwabach, Germany) at 200 rpm for 1 min. Then, Carbopol was slowly added dropwise at 250 rpm, and the speed was then increased to 800 rpm for 7 min to form a mixture with a gel appearance. Finally, the oil phase was added dropwise at 1200 rpm for 12 min.

#### 2.2.3. Physicochemical Characterization

The prepared emulsions were physiochemically characterized by assessing droplet-size distribution, rheological response, stability, and texture. Droplet-size distribution was evaluated using a MasterSizer^®^ 3000 (Malvern Panalytical, Malvern, UK), with refractive indices of 1.33 for water and 1.47 for the emulsion droplets. Rheological analysis, including viscosity, shear stress, storage modulus, and loss modulus, was performed using a Discovery Hybrid Rheometer HR-2 (TA Instruments, New Castle, UK) with plate geometry at 20 °C. A frequency sweep was conducted at 1% strain between 0.01–1 Hz with 10 points per decade. Flow curves were obtained using a frequency range of 0.1 to 100 Hz. Emulsion stability was assessed with the aid of a Turbiscan^TM^ LAB Stability Analyzer (Formulaction SA, L’Union, France) at a temperature of 25 °C, with a total of 10 sweeps per sample. The texture profile of the emulsions was evaluated with a TA.HD*plus*C Texture Analyzer (Stable Micro Systems, Godalming, UK) in compression-traction mode. Emulsions were loaded into 500 mL containers (90 mm diameter) at a height of 25 mm. A penetration test was carried out using a cylindrical probe P/35 (35 mm diameter, aluminum), which measured compression force at a rate of 2.0 mm/s [32].

#### 2.2.4. Biocompatibility Assays

##### Cell Culture

The HaCat cell line was provided by the Basic Medical Sciences Laboratory of the Faculty of Medicine of the Universidad de los Andes. The HaCat cells were cultured in DMEM supplemented with 10% FBS at 37 °C, 5% CO_2_, and 75% humidity. The medium was replaced three times per week until the cells reached 80% of confluence.

##### Cytotoxicity Assay

The assay was carried out according to the ISO 10993-5(C) standard. The cytocompatibility of the lavender essential oil, the CW49 peptide, the CW49–lavender emulsion, the lavender emulsion, and the control emulsion were assessed by measuring their impact on the metabolic activity of HaCaT human keratinocytes, with the aid of a colorimetric assay based on MTT. The lavender oil was evaluated at 1, 0.5, 0.25, and 0.125% *w*/*w*, the peptide was evaluated at 5, 10, 20, 50, 80, and 100 µg/mL, and the three emulsions were evaluated at 3, 1.5, 0.75, 0.375, and 0.187% *v*/*v*. The concentrations were diluted in non-supplemented DMEM medium. A total of 100 µL of cell-stock solution in DMEM medium supplemented with 10% FBS was seeded in a 96-well microplate at a cell density of 1 × 10^4^ cells/well. Microplates were incubated at 37 °C, 5% CO_2_, and a humidified atmosphere for 24 h. Then, DMEM medium supplemented with 10% FBS was removed and replaced with the different treatments. Cell viability was studied at 24 and 48 h post-exposure. To determine the viability percentage, 10 µL of MTT reagent was added to each well, and the microplates were then incubated under the same conditions for 2 h. Supernatants were subsequently discarded, and 100 µL of DMSO was added to each well to dissolve the formazan crystals. The absorbance was read at 595 nm in a microplate reader (Multiskan™ FC Microplate Photometer, Thermo Scientific, Waltham, MA, USA, FIN). The positive control comprised lysed cells using 10% (*v*/*v*) Triton X-100, while the negative control was non-supplemented DMEM medium.

##### Hemolysis

The assay was carried out by direct contact, as described in the ISO 10993-4 standard. The evaluated treatments were the control emulsion, the lavender emulsion, and the lavender–CW49-peptide emulsion. Initially, 2 × 10^7^ erythrocytes from a healthy human blood donor (informed consent obtained according to minute number 928-2018 of the Ethics Committee at the Universidad de los Andes) were centrifuged at 1800 rpm for 5 min and washed three times with NaCl 150 mM. Subsequently, buffer PBS (1X) was added to reach a 1:10 dilution. In a 96-well plate, 100 µL of the erythrocyte solution was added in triplicate to 100 µL of the treatments. The well plate was incubated at 37 °C and 5% CO_2_ for two hours. Finally, the well plate was centrifuged, the supernatant transferred to a brand-new well plate and the absorbance read at 450 nm. The positive control was Triton X-100 and the negative control was PBS (1X).

##### Platelet Aggregation

The assay was carried out by direct contact, as described in the ISO 10993-4 standard. The evaluated treatments were the control emulsion, the lavender emulsion, and the lavender–CW49-peptide emulsion. Initially, a human blood sample was centrifuged in sodium citrate tubes at 1000 rpm for 15 min to obtain platelet-rich plasma (PRP). In a 96-well plate, 100 µL of the emulsions were added to each well followed by carefully adding 70 µL of PRP. Exposure lasted for 3 min. The positive control was 6U thrombin, while the negative control was PBS (1X). Aggregation was measured by absorbance at 620 nm in a microplate reader (Multiskan™ FC Microplate Photometer, Thermo Scientific, FIN).

#### 2.2.5. Antibacterial Assay

The antibacterial activity of the emulsions was evaluated by agar diffusion assay and colony-forming-units count as described by Muñoz et al. [31]. Bacterial cells from *S. aureus* and *E. coli* were inoculated overnight, after which a colony was cultured in LB medium at 37 °C for four hours until a McFarland standard of 0.4 was reached. Next, 1 × 10^7^ CFU of the bacteria were exposed to 500 µL of the CW49–lavender emulsion, the lavender emulsion, and the control emulsion for 2 h at 37 °C. The exposed bacteria were serially diluted from 1 × 10^6^ CFU to 1 × 10^2^ CFU, and the final dilution was plated onto Mueller–Hinton agar in Petri dishes in triplicate. Following incubation at 37 °C for 18 h, CFUs were counted by CFU.Ai v1.4 (Medixgraph Inc., Fremont, CA, USA).

#### 2.2.6. 2D Wound-Healing Assay

To assess the regenerative potential of the CW49 peptide and the lavender oil, and to determine their optimal concentrations for inclusion in the emulsion formulation, a wound-healing assay was carried out in HaCat cells following the protocol established by Suarez et al. [33]. The peptide was tested at six concentrations: 5, 10, 20, 50, 80, and 100 µg/mL in DMEM supplemented with 10% FBS. The lavender essential oil was evaluated at three concentrations, 0.5, 0.25, and 0.125% *w*/*w*, in DMEM supplemented with 10% FBS. Once the optimal concentrations were determined (20 µg/mL for the CW49 peptide and 0.25% *w*/*w* for the lavender oil), the lavender, the control, and the lavender–CW49-peptide emulsions were synthetized, and wound-healing assays were performed. The emulsions were assessed at 2% *w*/*w* by the extraction method in DMEM supplemented with 10% FBS. HaCaT cells were seeded in a 24-well plate at a concentration of 1 × 10^5^ cells/well and cultured until reaching 80% confluence. Cell cultures were scratched with a 200 µL sterile pipette tip using a mold designed by A. Suarez et al. [33], and detached cells were washed away with PBS (1X). Then, 600 µL of different treatment concentrations were added to each well, with DMEM supplemented with 10% FBS serving as a control. Each treatment was performed in triplicate. It is important to note that horizontal reference lines were drawn at the bottom of each well on the 24-well plate using an ultrafine tip marker, providing a grid for alignment and consistent image acquisition. Images were captured at 0 h, 4 h, 8 h, 20 h, and 24 h under a Zeiss inverted microscope at a 10× objective.

Scratch area, width, and average standard deviation of the scratch width were obtained using the ImageJ/Fiji^®^ Version 2.3.0/1.53t Wound healing size tool plugin new version created by A. Suarez et al. [33]. The rate of cell migration (*R_M_*) and percentage of wound closure (*W_c_* %) were calculated according to Equations (1) and (2), respectively:

(1) $$R_{M}=\frac{W_{i}-W_{f}}{t}$$

(2) $$W_{c}\left(\%\right)=\frac{A_{t=0}-A_{t=∆t}}{A_{t=0}}*100\%$$

where *W_i_* is the average initial wound width, *W_f_* denotes the average final wound width (both in μm), and *t* is the assay duration in hours. Additionally, *A_t_*_=0_ is the initial wound area, and *A_t_*_=∆*t*_ is the wound area after *n* hours of the initial scratch, both measured in μm^2^.

#### 2.2.7. Statistical Analysis

To analyze the results of the wound-healing assay, one-way ANOVA and Bonferroni tests were used for multiple comparisons. The data were processed and plotted using GraphPad Prism software v9.

## 3. Results

The same 10:90 O/W% formulation with a surfactant ratio of 60:40 was used to manufacture three distinct emulsions, each containing the active ingredients of lavender essential oil at 0.25% *w*/*w* and CW49 peptide at 20 μg/mL. The chemical composition of the lavender essential oil was determined using GC-MS, followed by a physicochemical characterization to assess droplet-size distribution, frequency curves, flow curves, and stability. Biocompatibility and antibacterial tests were conducted on the three emulsions. The regenerative ability of the emulsions was demonstrated through a wound-healing assay. These findings provided valuable insights into the potential of these emulsions as a cost-effective and efficient wound-healing treatment.

### 3.1. Chemical Composition of the Lavender Essential Oil Using Gas Chromatography—Coupled to Mass Spectrometry (GC-MS)

The lavender essential oil purchased from Naturales Casvior (Bogotá, Colombia) and used in the emulsions was analyzed by GC-MS. This essential oil was extracted from the flowers of *Lavandula angustifolia*. The results showed that the lavender essential oil primarily consisted of sesquiterpenoids and oxygenated monoterpenoids like linalool, linalyl acetate, camphor, and 1,8-cineole. The complete composition is shown in Table 2. 

### 3.2. Physicochemical Characterization of the Emulsions

**Figure 1:**
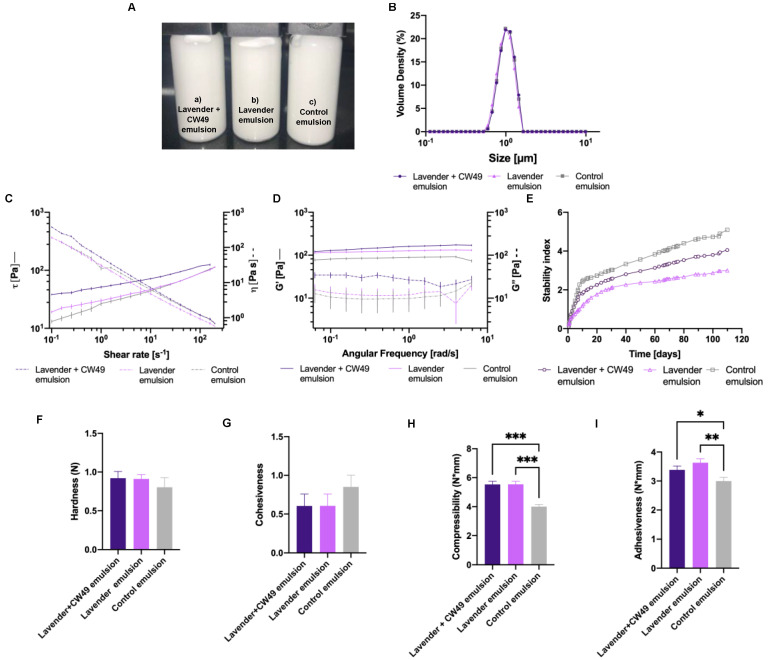
(**A**) (**a**) Lavender–CW49 peptide emulsion with a concentration of 0.25% *w*/*w* of essential oil and 0.2 µg/mL of CW49 peptide, (**b**) lavender emulsion with a concentration of 0.25% *w*/*w* of essential oil, and (**c**) control emulsion (without the active components). Rheological characterization: (**B**) droplet-size distribution of the emulsions. Mean size: lavender emulsion: 1.11 μm, lavender–CW49-peptide emulsion: 1.11 μm, and control emulsion: 1.08 μm. (**C**) Flow curve of stress τ vs. shear rate (continuous line) and viscosity η vs. shear rate (discontinous line) at 0.1–200 s^−1^. (**D**) Frequency sweep analysis of emulsions at 0.01–1 Hz, continuous lines correspond to storage modulus G′ and discontinuous lines correspond to loss modulus G″. (**E**) Stability of the emulsions by stability index. Textural analysis of the lavender–CW49-peptide emulsion, lavender emulsion, and control emulsions: (**F**) hardness, (**G**) cohesiveness, (**H**) compressibility, and (**I**) adhesiveness. (one-way ANOVA. (***) corresponds to *p* < 0.001, (**) to *p* < 0.01 and (*) *p* ≤ 0.05.

The 10:90% *w*/*w* (O/W) formulation was used to manufacture three different lavender emulsions: lavender–CW49 peptide with a concentration of 0.25% *w*/*w* of essential oil and 20 μg/mL of CW49 peptide (Figure 1A(a)), lavender emulsion with a concentration of 0.25% *w*/*w* of essential oil (Figure 1A(b)), and a control emulsion (without active components) (Figure 1A(c)). These emulsions were characterized by droplet-size distribution, frequency curves, flow curves, and stability as shown in Figure 1.

The droplet-size distribution D[4,3] indicates that the three emulsions exhibited a normal size distribution with sizes around 1.10 µm (Figure 1B). The flow curves of shear stress and viscosity (Figure 1C) demonstrated that viscosity decreased as the shear rate increased, and their shear stress increased as a function of the shear rate. These results are characteristic of a pseudoplastic fluid. The CW49–lavender emulsion exhibited higher viscosity and shear stress. Regarding the frequency curves obtained by the oscillatory sweep test, the emulsions showed a viscoelastic behaviour with their storage modulus higher than the loss modulus (Figure 1D). In general, the emulsions exhibited linear storage moduli (G′), within the same order of magnitude (~100 Pa), as a function of the angular frequency. On the other hand, the loss moduli (G″) showed a non-linear behaviour, oscillating around the same order of magnitude (~10 Pa). However, the CW49–lavender emulsion exhibited higher storage moduli (G′) and loss moduli (G″) than the other emulsions. The stability over the time was analyzed using the Turbiscan stability index (TSI) (Figure 1E); the emulsions remained stable during 120 days and exhibited similar behavior.

According to the texture-profile analysis, the CW49–lavender emulsion and the lavender-only emulsion demonstrated hardness values of approximately 0.92 N and 0.91 N, respectively. These values are notably higher than the control emulsion, which was measured at 0.8 N (Figure 1F). In contrast, the CW49-peptide–lavender emulsion and the lavender emulsion presented lower values of cohesiveness (around 0.60) compared to the control emulsion, as shown in Figure 1G. Finally, the CW49–lavender emulsion and the lavender emulsion exhibited significantly greater adhesiveness. In addition, the consistency index and the flow index of the three emulsions are reported in Table 3.

### 3.3. Biocompatibility Assays

**Figure 2:**
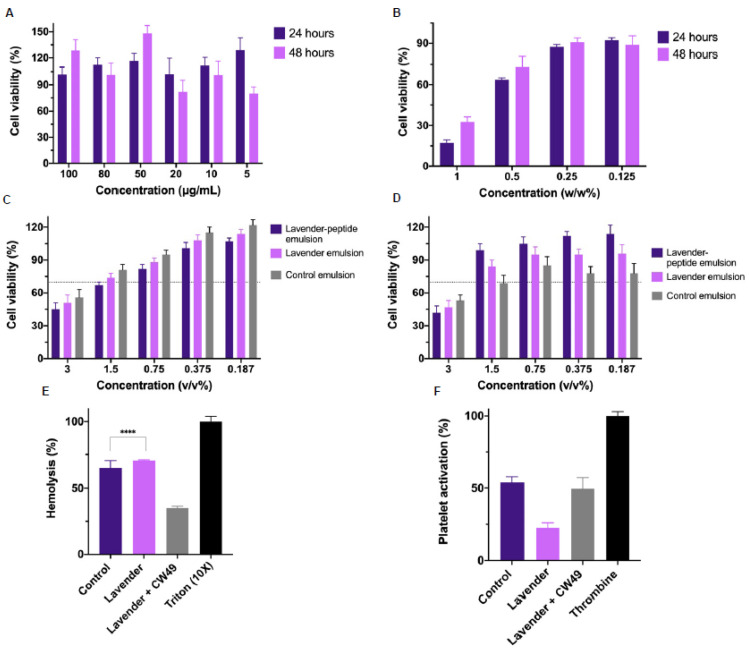
Cytotoxicity assays: (**A**) MTT assay results for the CW49 peptide (100, 80, 50, 20, 10, and 5 μg/mL) at 24 and 48 h. (**B**) MTT assay results for the lavender essential oil (1, 0.5, 0.25, and 0.125% *w*/*w*) at 24 and 48 h. (**C**) MTT assay results for the CW49–lavender emulsion, lavender emulsion, and control emulsion (3, 1.5, 0.75, 0.375, and 0.187% *v*/*v*) at 24 h. (**D**) MTT assay results for the CW49–lavender emulsion, lavender emulsion, and control emulsion (3, 1.5, 0.75, 0.375, and 0.187% *v*/*v*) at 48 h. Biocompatibility assays of the lavender–CW49-peptide emulsion with a concentration of 0.25% *w*/*w* of essential oil and 0.2 µg/mL of CW49 peptide, lavender emulsion with a concentration of 0.25% *w*/*w* of essential oil, and the control emulsion (without the active components). In the hemolysis assay (**E**), Triton X-100 was used as the positive control, while PBS 1X was the negative one. In the platelet aggregation assay (**F**), plasma rich in platelets (PRP) was employed as the negative control and Thrombin (6U) as the positive one. (****) corresponds to *p* < 0.0001.

To determine the appropriate concentrations of lavender oil and CW49 peptide for the topical emulsions, cytocompatibility was assessed using the MTT assay for 24 and 48 h in human keratinocytes (HaCaT). Figure 2A,B show the results for the peptide and the essential oil, respectively. At 24 and 48 h, all tested CW49-peptide concentrations (ranging from 100 µg/mL to 5 µg/mL) maintained viability. For the lavender essential oil, concentrations above 0.25% *w*/*w* showed viabilities above 70% after 24 and 48 h. Furthermore, the viability of the CW49–lavender emulsion, the lavender-only emulsion, and the control emulsion was evaluated using the MTT assay over 24 and 48 h periods. The emulsion concentrations varied from 3% *v/v* to 0.187% *v*/*v*. Generally, all three emulsions exhibited viability values below 70% after both 24 and 48 h, with viability increasing across consecutive concentrations.

Additionally, hemocompatibility assays were employed to characterize the developed emulsions without dilution, as these treatments are intended for topical application on the skin’s surface. According to the hemolysis assay (Figure 2E), both the control and the lavender emulsion exhibited hemolysis percentages above 50%, while the CW49–lavender emulsion had a hemolysis percentage of 25%. The platelet aggregation assay (Figure 2F) showed about 50% aggregation percentage for the CW49–lavender emulsion and the control emulsion (49.48% and 53.90%, respectively), and below 25% for the lavender emulsion.

### 3.4. Assesment of Antibacterial Activity of Lavander Emulsion

**Figure 3:**
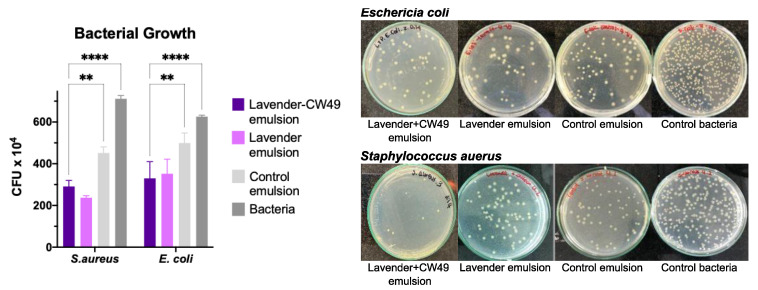
Antibacterial activity of the CW49–lavender emulsion, lavender emulsion, and control emulsion and bacteria (CFU × 10^4^) by agar diffusion assay and counts of colony-forming- units (CFUs) tested in *Escherichia coli* (*E. coli*) ATCC 25922, and *Staphylococcus aureus* (*S. aureus*) ATTC 23235. The positive control was bacteria grown in phospate buffer. (****) corresponds to *p* < 0.001 and (**) *p* < 0.01. Statistically significant differences were for *p* < 0.05.

The antibacterial activity of the CW49–lavender emulsion, the lavender emulsion, and the control emulsion was determined by agar diffusion assay and counts of colony-forming units (CFUs) against Gram-positive bacteria *Staphylococcus aureus* (*S. aureus*) and Gram-negative bacteria *Escherichia coli* (*E. coli*). Figure 3 shows the results at 10^4^ CFU. The CW49–lavender emulsion and lavender emulsion inhibited bacterial growth of *S. aureus* and *E. coli* by around 67% and 44%, respectively. In contrast, the control emulsion inhibited growth of Gram+(positive) and Gram-(negative) strains by 36% and 20%, respectively. Overall, the lavender emulsion exhibited better bacteriostatic activity against *S. aureus*.

### 3.5. Wound-Healing Assay

**Figure 4:**
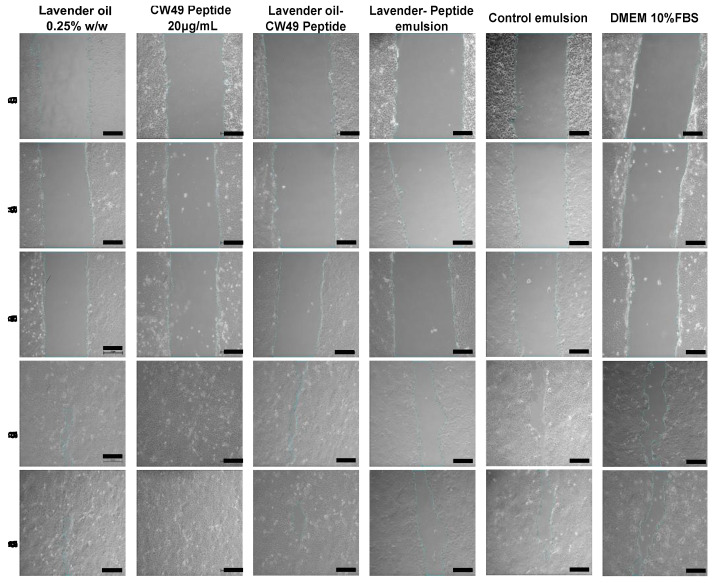
Images of the wound-healing assay to evaluate the regenerative potential in a monolayer of human keratinocyte cell line (HaCaT). The treatments evaluated were lavender essential oil at 0.25% *w*/*w*, CW49 peptide at 20 μg/mL, the two active compounds joined: lavender essential oil at 0.25% *w*/*w* and CW49 peptide at 20 μg/mL, the lavender–CW49-peptide emulsion, the control emulsion, and the control DMEM medium supplemented with 10% FBS. The time lapses were 0, 4, 8, 20, and 24 h (scale bar = 100 μm).

**Figure 5:**
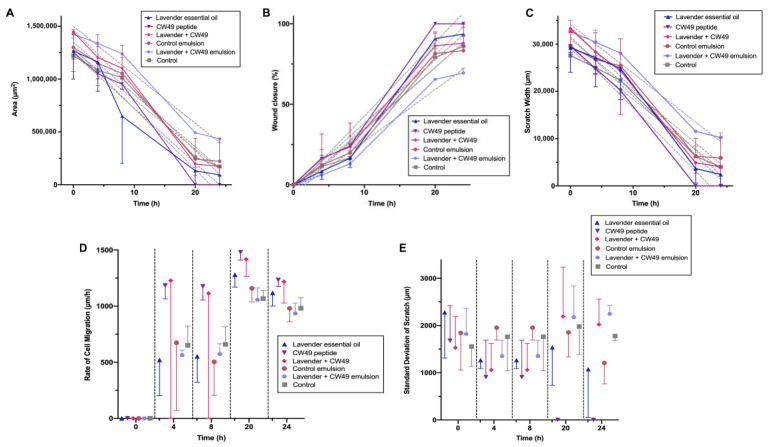
2D Wound-healing assay to evaluate the regenerative potential in a monolayer of human keratinocyte cell line (HaCaT) in a time lapse of 0, 4, 8, 20, and 24 h. The treatments evaluated were lavender essential oil at 0.25% *w*/*w*, CW49 peptide at 20 μg/mL, the two active compounds joined: lavender essential oil at 0.25% *w*/*w* and CW49 peptide at 20 μg/mL, the lavender–CW49-peptide emulsion, the control emulsion, and the control DMEM medium supplemented with 10% FBS. (**A**) Scratch area in μm^2^. (**B**) Wound closure in %. (**C**) Scratch width in μm. (**D**) Rate of cell migration in μ/hour. (**E**) Standard deviation of the scratch width in μm.

The results shown in Figure 4 and Figure 5 demonstrate that the CW49 peptide was highly effective in promoting closure in the 2D wound-healing assay for keratinocytes. The peptide’s effect on wound closure increased at a concentration of 20 µg/mL, as evidenced by the reduction in wound area and increase in wound-closure percentage. The peptide’s activity reached 100% closure after 20 h, as demonstrated in Figure 5A,B. The wound-closure percentages for the lavender essential oil, the lavender oil–CW49 peptide, DMEM 10% SBF, the control emulsion, and the lavender–peptide emulsion were 93.56%, 87.96%, 85.98%, 83.33%, and 69.4%, respectively. Furthermore, after 20 h, the scratch width in Figure 5C was significantly reduced, particularly in the presence of the CW49 peptide, with similar behavior observed in wound-closure percentage for the other treatments. Conversely, Figure 5D reveals that after 4 h, the rate of cell migration of cells exposed to the different treatments substantially increased, especially for those exposed to the CW49 peptide and the combination of the CW49 peptide and the lavender essential oil, which approached average values of 1479.7 µm/h and 1417.4 µm/h after 20 h of exposure, respectively. However, at the end of the experiment, the rates of cell migration for all treatments decreased. Finally, Figure 5E presents the standard deviation of the scratch width, which is likely an indicative of the heterogeneity of the scratch width, which may indicate the heterogeneity of the scratch width at each time point and suggest differences in how cells migrate. As expected, the width deviation does not appear to be a function of the evaluated times or the medium used and does not show a specific trend.

## 4. Discussion

Oil-in-water (O/W) emulsions are commonly used in topical treatments to combine hydrophilic and hydrophobic compounds in the same formulation. The incorporation of two active compounds aims to synergistically apply their respective benefits. As a result, the pharmaceutical industry frequently employs these emulsions to provide moisturizing properties to lotions without creating a sticky texture [39]. Moreover, these formulations should have appropriate mechanical and sensory properties to ensure product acceptability and efficacy. Surfactants are thus employed to stabilize the interaction between the two phases. In the emulsions developed in this study, the surfactant Tween 20 was used in the aqueous phase, and the surfactant Span 80 in the oil phase [40]. The hydrophilic–lipophilic balance (HLB) represents the ratio between the non-ionic surfactants soluble in water and oil. In this case, all prepared emulsions had an HLB of 11.74. This HLB falls within the acceptable range of 8–18 for stable oil-in-water emulsions [41,42]. Emulsion stability changes were analyzed using the Turbiscan instrument (Figure 1D), revealing no visible changes like coalescence or flocculation in the three emulsions, as TSI values remained below 5 after 109 days [39]. This stability can be attributed to the presence of Carbopol, which stabilizes the emulsion system by reducing the surface tension [43].

Droplet-size distribution was determined using the Brouckere diameter D[4,3] in a Mastersize 3000 instrument [44]. The emulsions prepared with the 10:90% *w*/*w* formulation exhibited a uniform size distribution, with sizes about 1.10 µm (Figure 1B), which is suitable for preventing instability phenomena [39]. Moreover, the droplet size can also be attributed to the presence of Carbopol, which prevents oil-droplet coalescence. The incorporation of Carbopol as a thickener in the continuous phase is crucial for providing stability, consistency, and droplet homogeneity [31,32]. Regarding rheological behavior, the emulsions exhibited shear-thinning and pseudoplastic properties (Figure 1C and Table 3). The flow curves of shear stress and viscosity in the 10:90% *w*/*w* emulsions demonstrated pseudoplastic behavior due to their resistance to structural rupture during exposure to a high shear rate. This type of fluid exhibits shear-thinning properties, as the apparent viscosity decreases when the shear rate increases. Still, this relationship is not linear, since it follows the power law [40].

Oil-in-water (O/W) emulsions frequently exhibit viscoelastic behavior, as evidenced by the frequency curves obtained from the oscillatory sweep test (Figure 1E). The three emulsions in this study showed a plateau zone in the storage moduli (G′), indicating an elastic response with the same order of magnitude (~100 Pa). The loss moduli (G″) showed non-linear values of ~10 Pa for the control emulsion and the lavender emulsions and ~50 Pa for the CW49–lavender emulsion, consistent with viscosity recovery after shear stress [32]. In terms of texture analysis (Figure 1F–I), the CW49–lavender, the control, and the lavender emulsions showed low hardness compared to some topical wound-healing applications [45,46,47]. However, topical treatments should ideally possess low hardness and high adhesiveness [45,46]. Increased hardness is related to increased formulation viscosity due to Carbopol’s role as a thickening agent [46]. Compressibility and adhesiveness parameters relate to ease of spreadability on the skin and bioadhesion, with the CW49–lavender emulsion and the lavender emulsion demonstrating greater adhesiveness than the control emulsion. Cohesiveness, which pertains to resistance to a second deformation [45], influences full structural recovery after application. In this case, the results align well with topical-application requirements [48].

Cytocompatibility assays, shown in Figure 2A,B, show the viability of human keratinocyte cells (HaCaT) after 24 and 48 h exposure to varying concentrations of CW49 peptide and lavender essential oil. Viability percentages ranged from approximately 150% to 85% for the peptide and from 30% to 90% for the lavender oil. The ISO 10993-5 standard accepts a viability percentage of 70% as non-cytotoxic [49]. In the case of the CW49 peptide, concentrations of 100, 80, and 50 µg/mL resulted in greater than 90% cell viability. The selected concentration for incorporation into the emulsion (20 µg/mL) exhibited an 85% viability, striking an optimal cost-benefit balance. For the lavender essential oil, Figure 2B demonstrates that at lower concentrations (0.25% *w*/*w* and 0.125% *w*/*w*), HaCat cells retained about 90% viability. In contrast, higher concentrations reduced cell viability to between 30 and 70%. The cytotoxic behavior is primarily attributable to the presence of linalool and linalyl acetate, key components of essential oil [50,51]. For this reason, the 0.25% concentration was deemed safe for the emulsion preparation and subsequently tested in the wound-healing assay. In addition, we evaluated the viability of three formulations: the CW49–lavender emulsion, the lavender-only emulsion, and the control emulsion. Viability percentages for these emulsions varied from 40% to 110%. This variability is attributed to the specific components of the emulsions, such as the surfactants Tween 20 and Span 80, which have fatty acid-based chemical compositions [52].

Hemocompatibility assays for the lavender–CW49-peptide emulsion, the lavender emulsion, and the control emulsion were carried out using the direct contact method. Hemolysis results showed that the lavender emulsion exhibited a higher hemolytic percentage (≈70.44%), attributable to the lavender essential oil and formulation components. Essential oils, known for their hydrophobic behavior, have been reported as hemolytic [53]. Linalool, the main component of lavender oil, causes erythrocyte lysis and is thus considered hemolytic [53,54]. Surfactants Tween 20 and Span 80 can also be hemolytic due to their fatty acid-based chemical characteristics, which damage erythrocyte membranes [52,55]. The presence of the CW49 peptide in the lavender–CW49-peptide emulsion reduced the hemolytic effect by 20%, which is comparable to commercial products like silver sulphadiazine, which is frequently used [56,57].

Furthermore, the platelet-aggregation assay (Figure 2D) shows that the emulsions exhibit platelet-aggregation properties. Consequently, the CW49–lavender-oil emulsion acts as an activating agent, aiding in the hemostasis phase of the wound-healing process. Specifically, it promotes the recruitment of platelets to interact with collagen and form a blood clot rich in fibrin [58]. Cell therapies, such as platelet-rich plasma (PRP) therapy, have been employed for chronic non-healing ulcers [59]. Therefore, this result supports the overall aim of the emulsion, which is to facilitate wound closure and promote the healing process.

To confirm the antibacterial activity of lavender essential oil, an antibacterial-activity assay was carried out by agar diffusion against *E. coli* and *S. aureus* (Figure 3) in the CW49–lavender emulsion, the lavender emulsion, and the control emulsion. The CW49–lavender emulsion and the lavender emulsion inhibited the growth of both bacterial strains, thus demonstrating bacteriostatic behavior. Research suggests that the antibacterial activity of essential oils results from the action of monoterpene and sesquiterpene components on the cell membrane [54]. In particular, lavender oil can disrupt membranes and cause bacterial-cell shrinkage due to its lipophilic components, leading to bacterial death [60]. Moreover, the lavender oil showed potent antibacterial activity against the Gram-positive bacterium *S. aureus*, likely due to the composition of the cell wall [61]. Based on these findings, the lavender essential oil was chosen as an active compound for the emulsion to control bacterial infections in skin wounds.

To assess the regenerative potential of the active compounds and the developed emulsions, a wound-healing model was performed on a 2D cell layer of human keratinocytes. The lavender oil, the CW49 peptide, a combination of lavender oil–CW49 peptide, the lavender–peptide emulsion, and the control emulsion were evaluated in this model (Figure 4 and Figure 5). Exposing keratinocytes to the CW49 peptide promoted migration and wound healing, consistent with previous studies due to the upregulation of angiogenic proteins, such as hypoxia-inducible factor (HIF)-1, endothelial nitric oxide synthase (eNOS) and inducible nitric oxide synthase (iNOS), and the inhibition of excessive inflammation [19]. Notably, H. Liu et al.’s study conducted an in vivo evaluation of the CW49 peptide in mice, determining that the CW49 peptide promotes wound healing with full-thickness dermal wounds in both normal and diabetic animals. Specifically, a thin layer of granulation tissue and a rapid re-epithelialization were observed following the application of the CW49 peptide [19]. These findings confirmed that the CW49 peptide, at a concentration of 20 µg/mL, was the active compound promoting wound healing in the proposed emulsion. In addition to its tissue-regeneration properties compared to the control, it also represents an appropriate cost–benefit ratio, given its relatively low concentration.

In the 2D wound model of a monolayer of the HaCaT cell line, the lavender essential oil demonstrated regenerative activity, albeit to a lesser extent than the CW49 peptide. Lavender oil accelerates granular tissue formation through collagen synthesis, tissue remodeling via collagen replacement from type III to type I, and wound shrinkage [29]. Other authors have also reported anti-inflammatory properties of lavender oil [25], which were confirmed in an in vivo wound model in mice, where no edema, discharge, or local infection was observed [62]. Therefore, this active component aids wound healing by accelerating closure and reducing inflammation. Nevertheless, the combination of both active components in the proposed emulsion did not show a significant difference compared to each component individually. Finally, the extracts of the lavender–peptide emulsion and the control emulsion showed a decrease in regenerative potential in the model, because the skin model was a 2D HaCaT cell monolayer, not on dermis-specific fibroblast cells that may differ in response. Cytotoxicity MTT assays of commercial products such as acticoat^TM^ and flamazine^TM^ have been reported using HEK and NHF cell lines, where viability was reduced by more than 40% [63]. Consequently, a full-skin 3D structure should be considered to evaluate topical treatments like emulsions.

## 5. Conclusions

In this study, we explored the in vitro wound-healing potential of emulsions based on lavender essential oil and the CW49 peptide, focusing on their physicochemical properties, biocompatibility, antibacterial activity, and wound-healing efficacy. The formulated emulsions exhibited pseudoplastic fluid behavior, uniform droplet-size distribution, and high stability over 120 days, with a texture profile suitable for topical treatments. Biocompatibility assessment of the active components and the emulsion revealed non-cytotoxic behavior for both the CW49 peptide at 20 μg/mL and lavender essential oil at 0.25% *w*/*w* in the viability assay using human keratinocyte (HaCaT) cells. Hemocompatibility assays demonstrated that the emulsions were hemolytic and aggregating, promoting the wound-healing process. Lavender oil exhibited antibacterial activity in the CW49–lavender emulsion and the lavender emulsion, making it an appropriate candidate for treating bacterial infections in wounds.

The wound-healing assay results indicated that the emulsion showed promising wound closure after 24 h of application, highlighting its regenerative potential. Hence, the proposed emulsions exhibit optimal characteristics for both regenerative and antibacterial applications. Specifically, we developed and evaluated a stable emulsion based on lavender oil and the CW49 peptide, focusing on its physicochemical and biological properties. Furthermore, we assessed its regenerative capacity in 2D monolayers of human keratinocyte cells, with results indicating promising potential for wound care. However, it is crucial to consider the tradeoffs and challenges associated with different factors impacting the wound-healing potential of these emulsions. For instance, a more complex and representative in vitro model, such as a 3D skin model or an ex vivo human-skin explant, should be utilized to validate the emulsion’s efficacy further. In vivo studies using animal models are also essential to assess the emulsion’s safety, tolerability, and effectiveness in a physiological context.

## Figures and Tables

**Table 1:** Composition of the (O/W) emulsions incorporating lavender oil and the CW49 peptide.

Emulsion	Control	Lavender	Lavender–CW49 Peptide
Composition (% *w*/*w*)			
Oily phase			
Lavender oil	-	0.25	0.25
Span 80	1.60	1.60	1.60
Mineral oil	8.40	8.15	8.15
Aqueous phase			
CW49 peptide	-	-	0.20
Tween 20	2.40	2.40	2.40
Carbopol	0.3	0.3	0.3
Triethanolamine	0.6	0.6	0.6
Water	86.7	86.7	86.5

**Table 2:** Retention times (t_R_), tentative identification, and relative amount (%) of the components of the volatile fraction present in the lavender-essential-oil sample analyzed by gas chromatography coupled to mass spectrometry (GC/MS) (full scan), and the range of relative amount reported.

t_R_ (min)	Tentative Identification	Relative Amount	Range of Relative Amount Reported [34,35,36,37,38]
16.3	α-Pinene	0.3	0.19–0.9
17.1	Camphene	0.2	0.32–0.6
18.3	β-Pinene	0.4	0.76–0.82
18.7	β-Myrcene	0.4	0.12–1.8
19.7	Hexyl acetate	0.4	0.3–0.4
20.3	p-Cymene	1.9	0.6–5.15
20.5	Limonene	1.5	0.15–8.5
20.7	1,8-Cineole	8.5	2.2–13.74
22.3	Cis-Linalool oxide	0.1	0.83–6.63
22.9	Trans-Linalool oxide	0.1	2.1–5.34
23.5	Linalool	32.6	17.10–49.50
24.3	Fenchol	0.2	-
24.8	1,2-Dihydrolinalool	0.3	-
25	p-Menth-3-en-1-ol	0.2	-
25.5	Camphor	9.5	0.9–19.9
26.1	Isoborneol	0.3	2.57–6.9
26.2	p-Menth-8-en-1-ol	0.1	0.06–5.21
26.4	Endo-Borneol	0.6	-
27.3	α-Terpineol	2.8	1.16–4.20
27.4	γ-Terpineol	1.1	-
28.1	Fenchyl acetate	0.2	-
29.2	Linalyl acetate	27.8	11.23–28.20
32.7	α-Terpinyl acetate	0.1	-
33	Neryl acetate	0.2	0.38–2.02
33.8	Geranyl acetate	3.5	0.26–2.85
35.8	trans-β-Caryophyllene	2.7	0.21–6.85
37	α-Humelene	0.2	-
41	Caryophyllene oxide	0.3	0.19–5.12

**Table 3:** Rheology indexes: consistency index (m) and flow index (n).

Emulsion	m	n
CW49 peptide–lavender	53.17	0.263
Lavender	31.24	0.244
Control	34.67	0.263

## Data Availability

Not applicable.
